# Growth cone-specific functions of XMAP215 in restricting microtubule dynamics and promoting axonal outgrowth

**DOI:** 10.1186/1749-8104-8-22

**Published:** 2013-12-01

**Authors:** Laura Anne Lowery, Alina Stout, Anna E Faris, Liya Ding, Michelle A Baird, Michael W Davidson, Gaudenz Danuser, David Van Vactor

**Affiliations:** 1 Department of Cell Biology, Harvard Medical School, Boston, MA, 02115, USA; 2 National High Magnetic Field Laboratory, Florida State University, Tallahassee, FL, 32310, USA

**Keywords:** XMAP215, TOG, Microtubule dynamics, Growth cone, Quantitative imaging, Cytoskeleton, Actin

## Abstract

**Background:**

Microtubule (MT) regulators play essential roles in multiple aspects of neural development. *In vitro* reconstitution assays have established that the XMAP215/Dis1/TOG family of MT regulators function as MT ‘plus-end-tracking proteins’ (+TIPs) that act as processive polymerases to drive MT growth in all eukaryotes, but few studies have examined their functions *in vivo*. In this study, we use quantitative analysis of high-resolution live imaging to examine the function of XMAP215 in embryonic *Xenopus laevis* neurons.

**Results:**

Here, we show that XMAP215 is required for persistent axon outgrowth *in vivo* and *ex vivo* by preventing actomyosin-mediated axon retraction. Moreover, we discover that the effect of XMAP215 function on MT behavior depends on cell type and context. While partial knockdown leads to slower MT plus-end velocities in most cell types, it results in a surprising increase in MT plus-end velocities selective to growth cones. We investigate this further by using MT speckle microscopy to determine that differences in overall MT translocation are a major contributor of the velocity change within the growth cone. We also find that growth cone MT trajectories in the XMAP215 knockdown (KD) lack the constrained co-linearity that normally results from MT-F-actin interactions.

**Conclusions:**

Collectively, our findings reveal unexpected functions for XMAP215 in axon outgrowth and growth cone MT dynamics. Not only does XMAP215 balance actomyosin-mediated axon retraction, but it also affects growth cone MT translocation rates and MT trajectory colinearity, all of which depend on regulated linkages to F-actin. Thus, our analysis suggests that XMAP215 functions as more than a simple MT polymerase, and that in both axon and growth cone, XMAP215 contributes to the coupling between MTs and F-actin. This indicates that the function and regulation of XMAP215 may be significantly more complicated than previously appreciated, and points to the importance of future investigations of XMAP215 function during MT and F-actin interactions.

## Background

A fundamental question in early neural development is how cytoskeletal dynamics are regulated to control axon outgrowth and navigation [1]. MTs, in particular, play a significant role in the neuronal growth cone during axon outgrowth [2]. They are necessary for axon elongation, axonal transport, and accurate steering of the growth cone. Despite their importance, only a few studies have examined the regulation of MT dynamics within living growth cones [3-5]. To identify MT regulators that are required within the growth cone, we previously performed genetic and proteomic screens in *Drosophila* and showed that Msps, ortholog of the conserved XMAP215/Dis1/TOG family, plays a significant role during embryonic axon guidance [6]. This protein family has received prominent attention in recent years as critical regulators of MT polymerization [7,8]. The founding member, XMAP215, was originally identified as a MT-associated protein from *Xenopus laevis* egg extracts that promotes MT assembly *in vitro*[9]. More recently, reconstitution assays and single-molecule imaging combined with structure-function analyses have provided useful insights into the mechanism by which XMAP215 catalyzes MT polymerization *in vitro*[10,11]. However, there have been few studies of XMAP215 and its family members *in vivo*[12-14], and none have examined its role(s) specifically within the neuronal growth cone.

In this study, we use quantitative analysis of high-resolution live imaging to examine the function of XMAP215 in embryonic *Xenopus laevis* neurons. We demonstrate that XMAP215 is required for persistent axon outgrowth *in vivo* and *ex vivo* by preventing axon retraction. Moreover, we discover that partial knockdown of XMAP215 leads to an unexpected increase in MT plus-end velocities selective to growth cones. We use MT speckle microscopy to determine that differences in overall MT translocation are a major contributor of this velocity change. Together, our data suggests that XMAP215 functions as more than a simple MT polymerase and is also likely involved in the coupling of MT-F-actin linkages.

## Results and discussion

### XMAP215 prevents spontaneous actomyosin-mediated axon retraction

**Figure 1:**
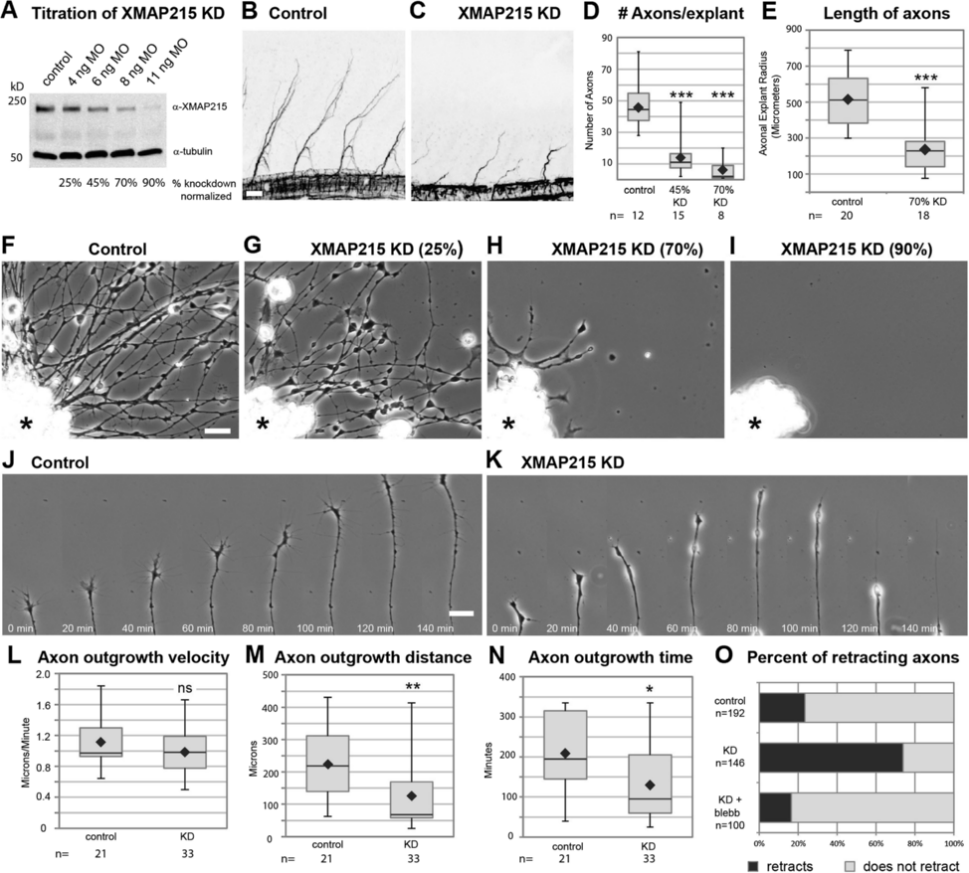
**Knocking down XMAP215 leads to reduced axon outgrowth due to increased rates of axon retraction. (A)** Western blot showing knockdown of XMAP215 with increasing levels of morpholino oligonucleotide (MO). **(B-C)** Confocal images of whole-mount embryo immunostaining for acetylated tubulin shows peripheral axon outgrowth at two days post-fertilization. **(D-E)** Quantification of axon outgrowth in culture after XMAP215 knockdown (KD). n = explant number **(D)**, axon number **(E)**. **(F-I)** Phase contrast images of axons from control or XMAP215 KD neural explants (*) after 24 hours of culturing. **(J-K)** Timelapse montage of representative axons. (See Additional file 1). **(L-O)** Quantification of axon outgrowth parameters from timelapse imaging, between 18 and 23 hours of culturing. Box-and-whisker plots indicate the mean (diamond), median, extrema and quartiles. *P < 0.05, **P < 0.01, ***P < 0.001 comparing KD with control. ns not significant. n = axon number. Bar is 50 μm for **(B,C)**, 20 μm for **(F-K)**.

To investigate the function of XMAP215 during vertebrate nervous system development, we inhibited its translation in *Xenopus laevis* embryos by utilizing an antisense morpholino oligonucleotide (MO) (Figure 1A). By two days post-fertilization, control embryos have entered a period of rapid nervous system development and axon outgrowth, but knocking down XMAP215 approximately 70% substantially reduced normal axon outgrowth *in vivo* (Figure 1B,C). To explore the mechanism that led to this reduced outgrowth, we examined the effect of XMAP215 knockdown (KD) on embryonic axons at higher resolution by culturing neural explants *ex vivo*[15]. We first determined how axon outgrowth parameters quantitatively change with varying levels of XMAP215 KD by performing a titration series with antisense MO. As levels of XMAP215 were reduced, the percentage of explants showing normal parameters of axon outgrowth steadily decreased (Figure 1D-I), with deficits in both axonal number and length. While 90% knockdown led to an absence of axon outgrowth (Figure 1I), consistent with the expected involvement of MT polymerization in this process [16,17], 70% knockdown allowed for some outgrowth but still resulted in a robust reduction of axonal length (Figure 1H). Thus, such intermediate dose allowed us to relate XMAP215 activity to axon outgrowth regulation by analyzing partial deficiencies while complete knockdown of this protein would simply break the system without offering insight into the normal XMAP215 function. For the remainder of experiments, we utilized 70% knockdown.

Given that XMAP215 is the only known MT polymerase [7], and as it is well-established that axon outgrowth requires polymerized MTs [17], the conventional view would suggest that diminished axogenesis was a result of slower outgrowth velocity due to reduced MT polymerization. However, timelapse imaging demonstrated that axon outgrowth velocities after XMAP215 KD were not significantly different from controls (Figure 1J-L, Additional file 1). Rather, there was a substantial reduction in the distance and time of persistent axon outgrowth prior to spontaneous retraction and a concomitant increase in the percentage of axons that retracted (Figure 1M-O). As axonal retraction normally results from forces mediated by non-muscle myosin II [18,19], we therefore asked whether inhibiting these forces would have an effect on the XMAP215 KD retraction phenotype. Indeed, we observed that axon retraction could be rescued by treating the XMAP215 KD axons with the myosin II inhibitor blebbistatin (Figure 1O). This suggests that XMAP215 is part of the machinery that normally allows for microtubules within the axon shaft to oppose retraction. It is well-known that in order for MTs to oppose the retractive forces that occur within axons, MTs must be functionally linked to actin, and dynein forces are essential in mediating this linkage [18,19]. As we observed that MTs are unable to oppose the contractile forces when XMAP215 levels are reduced, this implicates XMAP215 as another important player in the axonal MT-actin relationship.

### Reducing XMAP215 levels leads to increased MT plus-end velocities in growth cones

**Figure 2:**
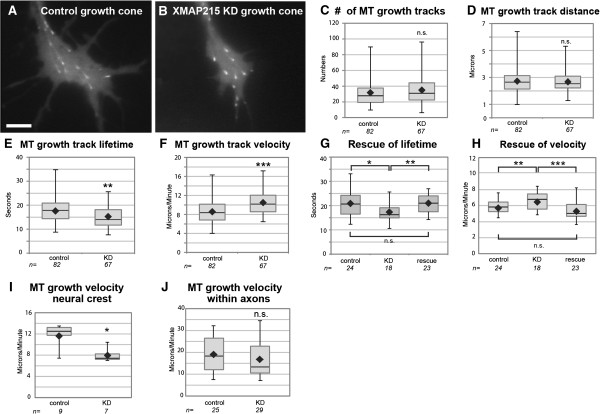
**Reducing XMAP215 function leads to an increase in microtubule (MT) plus-end velocity in growth cones. (A-F)** Data and quantitative analysis of the mean values per growth cone of EB1-GFP comets in control and XMAP215 knockdown (KD) (see Additional file 2). **(G, H)** Quantification of MT growth track parameters after adding XMAP215 mRNA to KD. **(I-J)** Quantification of MT plus-end growth velocities in neural crest and axons. Box-and-whisker plots indicate the mean (diamond), median, extrema and quartiles. *P < 0.05, **P < 0.01, ***P < 0.001, ns not significant. N = growth cone/cell number. Bar is 5 μm.

Since current models regarding XMAP215-family protein function focus primarily on its role as a MT polymerase, we next sought to examine whether specific MT dynamics were disrupted after XMAP215 KD. To test this, we quantified global parameters of MT dynamics using the Matlab-based open-source software plusTipTracker [20], following acquisition of high-resolution live images of tagged EB1, which binds all growing MT plus-ends [21]. Upon comparing XMAP215 KD and control growth cones, there were no obvious differences in terms of EB1 comet morphology, number of MT growth tracks, or average MT growth track distance (that is, the length of persistent forward MT polymerization) (Figure 2A-D, Additional file 2), suggesting that these MT parameters are not affected by 70% reduced levels of XMAP215 in growth cones. Consistent with a role for XMAP215 in promoting MT polymerization, growth track lifetime was significantly shortened after XMAP215 KD (Figure 2E). However, in contrast to conventional expectation, we discovered that MT growth track velocity in the knockdown was greater than in controls (Figure 2F). This 20% velocity increase was both significant and highly reproducible (occurring in 7/7 separate experiments, totaling data from 149 growth cones and 10,160 MT growth tracks). A second non-complementary antisense MO to XMAP215 showed an identical phenotype (not shown), suggesting that this velocity increase was not due to off-target effects. Moreover, the velocity and lifetime defects could be rescued by adding back XMAP215 (Figure 2G,H), confirming the phenotype specificity.

None of the XMAP215 functions described from *in vitro* studies would predict an increase in plus-end velocity after knockdown (for example, [10]). Therefore, we hypothesized that depletion of XMAP215 in our experiments affected a yet unexplored *in vivo* function. To address this, we examined EB1 fused to green fluorescent protein (GFP) dynamics in additional embryonic cell types, neural crest cells (a mesenchymal, multipotent cell type from dorsal neural tube) and epidermal cells. In contrast to the growth cone phenotype, knockdown of XMAP215 in these cells reduced MT plus-end velocities (Figure 2I, and not shown). Thus, while depleting XMAP215 in other cell types led to a phenotype consistent with XMAP215 functioning primarily as a MT polymerase, its effect in neuronal growth cones was quite different.

XMAP215 function in growth cones had never before been examined, although two studies showed that knockdown of XMAP215 orthologs led to reduced EB1-GFP velocities in axons [12,22]. Therefore, to verify our observations and to determine if *Xenopus* spinal cord neurons are atypical of other species, we compared MT dynamics in axons to growth cones. Here, we observed that EB1-GFP comets did not show the increase in velocity after XMAP215 reduction, and instead there was a trend towards slower velocities (Figure 2J), consistent with the axonal results from other organisms. This demonstrates that by looking in the growth cone compartment, we uncovered a new function of XMAP215 that is specific to the growth cone.

### XMAP215 has a MT lattice-binding function

**Figure 3:**
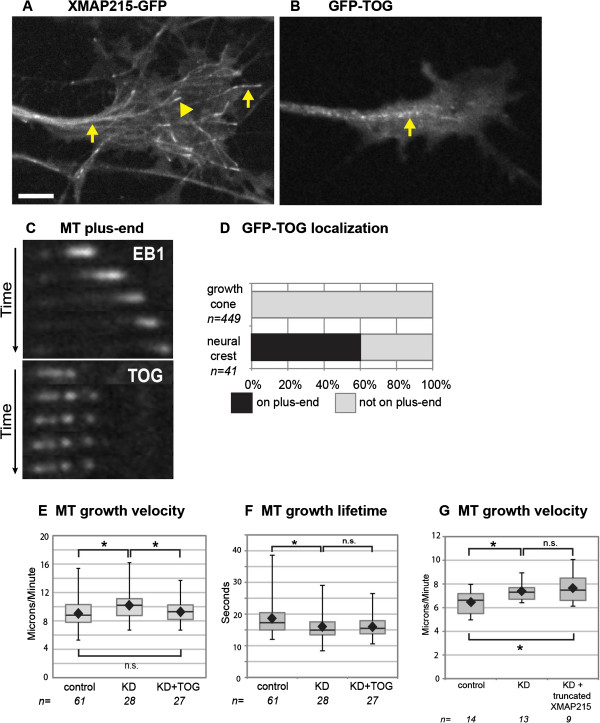
**GFP-TOG primarily localizes to microtubule (MT) lattice yet rescues MT plus-end velocity defect of XMAP215. (A)** Micrograph of XMAP215-GFP in neuron, with arrowhead pointing to plus-end tracking and arrows to lattice-binding. (See Additional file 3A) **(B)** Micrograph of GFP-TOG in neuron, with arrow pointing to lattice-binding. (See Additional file 3B) **(C)** Timelapse montage of representative MT, with mKate2-EB1 (top) and GFP-TOG (bottom). (See Additional file 3C) **(D)** Quantification of GFP-TOG localization in growth cones and neural crest. n = number MTs examined. **(E-G)** Quantification of MT parameters. Box-and-whisker plots indicate the mean (diamond), median, extrema and quartiles. *P < 0.05 comparing conditions. ns not significant. n = axonal growth cone number. Bar is 10 μm for **(A-B)**, 1 μm for **(C)**.

To determine how XMAP215 might restrict plus-end velocity specifically in growth cones, we first tested whether XMAP215, which tracks MT plus-ends (Figure 3A, Additional file 3A), might be directly inhibiting MT polymerization in growth cones, either by acting as a MT depolymerase, as has been seen in some *in vitro* systems under specific conditions [7,23,24], or by competing with other + TIPs at the plus-end that promote more rapid MT polymerization in neurons. We investigated whether TOG, an XMAP215 ortholog that does not detectably track plus-ends in *Xenopus* growth cones (Figure 3B-D, Additional file 3B-C), could rescue the XMAP215 KD phenotypes. Surprisingly, GFP-TOG was able to rescue the plus-end velocity defect (Figure 3E). However, it was unable to rescue the shift in MT lifetime associated with XMAP215 KD (Figure 3F), which is consistent with a requirement of plus-end localization for driving persistent polymerization. Thus, this data suggests that MT plus-end velocity and MT lifetime parameters are separable. While MT growth track lifetime depends upon plus-end binding of XMAP215, MT plus-end velocity can be affected by an XMAP215/TOG function unrelated to plus-end binding.

What could be the non-plus-end function of XMAP215 in growth cones that accounts for the velocity change? Given the current understanding of XMAP215 activity, one simple hypothesis was that XMAP215, which contains multiple tubulin dimer-binding TOG domains, might normally sequester tubulin dimers in growth cones, thus restricting the cytoplasmic pool of tubulin available for polymerization. In this scenario, when XMAP215 levels are partially reduced, increased levels of free tubulin in the cytoplasm would kinetically drive faster polymerization. Consistent with this possibility, levels of XMAP215 protein in neural tissue were four times higher than non-neuronal cell types at the same developmental stage (not shown) [25]. Thus, in growth cones, an increased amount of XMAP215 might act as a tubulin dimer-binding sink. The efficiency of such a sink could be controlled rapidly depending on the growth cone signaling state. This might also explain why GFP-TOG, which can bind tubulin dimers, rescues the velocity defect, even though it does not track the plus-ends.

To test this tubulin sequestration model, we expressed a truncated version of XMAP215, which contains two TOG domains capable of binding tubulin dimers but does not bind MTs. However, in contrast to wild-type XMAP215, expression of the TOG domain construct was unable to rescue the plus-end velocity defect of XMAP215 KD (Figure 3G). Thus, a model whereby cytosolic XMAP215 sequesters tubulin dimers cannot be correct. With a simple sequestration model ruled out, the question of whether MT lattice-binding itself is central to the XMAP215/TOG function within the growth cone became more attractive. Not only GFP-TOG but also full-length XMAP215-GFP localize abundantly to the MT lattice, in addition to tracking the plus-ends (Figure 3A, Additional file 3A) [26,27].

Despite its lattice-binding *in vivo*, almost all studies of XMAP215 have been focused on its plus-end activity. However, there is precedence for + TIPs playing essential roles both as a plus-end tracker and as a MT lattice-binder in growth cones. For example, the + TIP CLASP was discovered to track plus-ends in the cell body of migrating epithelial cells but to decorate the entire MT lattice in leading edge lamella [28], and these dual modes of binding are due to differential regulation by upstream glycogen-synthase-kinase-3 signaling [29]. More recently, it was determined that these dual localizations of CLASP occur in growth cones, and that they regulate axon outgrowth in an opposing manner [3]. While CLASP plus-end tracking supports axon outgrowth, lattice-binding mediates axon growth inhibition by restricting MT protrusion into the periphery. This suggests the intriguing possibility that dual modes of XMAP215 binding to MTs may function similarly to differentially regulate MT dynamics. Given that we originally identified an XMAP215-family protein in a genetic and proteomic screen for CLASP interactors [6], dual modes of function based on differential binding may be a common mechanism that links these two + TIPs.

### XMAP215 affects MT translocation rates in growth cones

**Figure 4:**
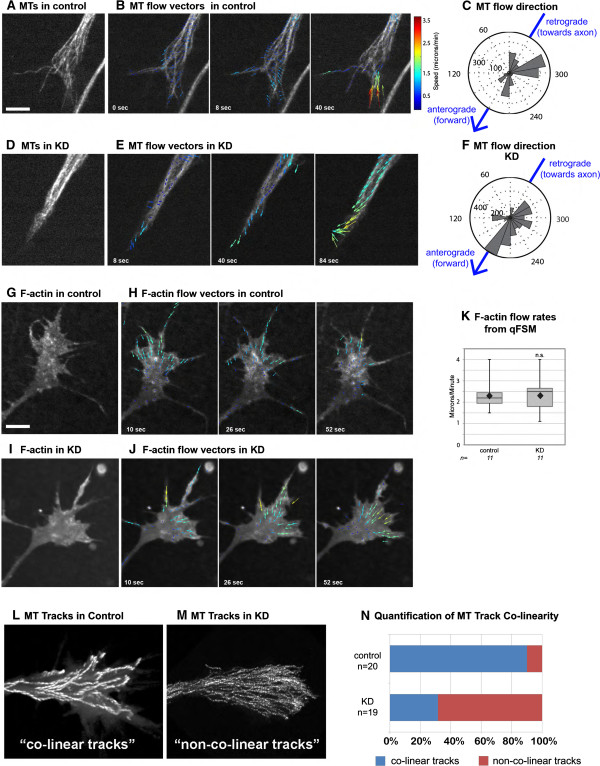
**XMAP215 knockdown (KD) leads to changes in microtubule (MT) lattice and F-actin flow rates. (A-C)** Micrograph of mKate2-tubulin at low levels in control growth cone **(A)**, overlaid with flow vectors calculated by QFSM software at three different time points **(B)**, and **(C)** rose plot of MT flow directions within all control growth cones, thresholded to include only vectors above the mean, in control. Zero degrees denotes flowing in the retrograde direction, towards the axon, while 180 degrees corresponds to the anterograde direction, with MTs flowing outwards in the direction of new growth (direction of arrow). (See Additional file 4A) **(D-F)** The same type of data as in **(A-C)**, but for XMAP215 KD growth cones. (See Additional file 4B) **(G-J)** Micrograph of F-actin labeled by fluorescent kabC in control growth cone **(G)** and XMAP215 KD **(I)**, with overlay of F-actin flow vectors from QFSM software at three different time points **(H,J)**. (See Additional file 4C,D) **(K)** Quantification of F-actin speckles using QFSM software. Note that wild-type flow rates are slower than traditional kymograph-measured F-actin retrograde flow rates because manual analysis tends to selectively measure prominent, fast-flowing speckles, while QFSM measures every F-actin speckle within the entire growth cone. For analysis of F-actin flow quantification, only the top quartile of flow vectors for each movie was utilized. This excludes any false positive speckle detection from analysis, and also enhances the likelihood of identifying differences in maximum rates of F-actin retrograde flows. **(L-N)** Micrograph overlays of EB1-GFP tracks from a two-minute timelapse image series in control **(L)** and XMAP215 KD **(M)**. **(N)** Quantification of the percentages of growth cones with co-linear versus non-co-linear tracks. Box-and-whisker plots indicate the mean (diamond), median, extrema and quartiles for each group. ns not significant. n = number of growth cones examined. Scale bar is 5 μm.

In considering how lattice-binding may affect MT dynamics, we reasoned that XMAP215 KD might lead to changes in overall MT network movements. To examine this, we used quantitative fluorescent speckle microscopy (QFSM), in which speckles of fluorescent-tubulin complexes incorporate into the polymer network and serve as points of reference for tracking the MT network [30,31]. This shows that, in wild-type growth cones, the general trend of MT movement is in the retrograde direction, especially in the periphery of the growth cone (Figure 4A-C, Additional file 4A). This was expected due to coupling of MTs to F-actin retrograde flow, which is highest in the periphery [32]. However, even in wild-type, MT movements continuously change directions throughout the growth cone (Figure 4B), suggesting frequent transient decoupling of MTs from F-actin retrograde flows.

After XMAP215 KD, we discovered that there was an increase in MT translocations in the anterograde direction (Figure 4D-F, Additional file 4B). Outward MT lattice movement must be a contributing factor for the faster plus-end velocities after XMAP215 KD, as reducing backward MT movement would lead to increased measured EB1 velocities. It was unclear, however, whether increased MT translocations outward were due to enhanced decoupling of MTs from F-actin retrograde flow or to a significant reduction in F-actin retrograde flow itself. Thus, we examined F-actin flow rates using QFSM of F-actin (Figure 4G). Unlike MT flows, F-actin network flows are significantly more homogeneous, with virtually all F-actin speckles moving in the retrograde direction (Figure 4H, Additional file 4C). This was also the case after XMAP215 KD, with no repetitive flow directional changes (Figure 4I-J, Additional file 4D). While we did observe a reduction in F-actin flow rates in some of the XMAP215 KD growth cones, especially within the lamella (not shown), this result was not consistent, and overall there was no statistical difference between F-actin retrograde flow in control and XMAP215 KD (Figure 4K). Thus, a reduction in F-actin retrograde cannot account for the 2 μm per minute increase in EB1 velocity that we measured after XMAP215 KD. Rather, the logical explanation for the increased MT flow outwards is an enhanced transient decoupling of MTs from the F-actin network. We speculate that as decoupling increases in the XMAP215 KD, MTs become more permissive to the action of MT motors which are normally needed to push the MTs against the F-actin retrograde flow [33,34], thus sliding the MT lattices forward.

In addition to normally constraining MT forward translocation, the F-actin array also provides a guide map for MT exploration of the growth cone [1]. In particular, MTs tend to follow each other along the same paths, and these correspond to locations of F-actin bundles [35-37]. Consistently, when we examined MT trajectories in control growth cones, we observed a high rate of co-linear tracks (Figure 4L,N). However, in the XMAP215 KD, there was a striking increase in the number of growth cones with non-co-linear tracks (Figure 4N), whereby MT trajectories were more randomly distributed throughout the entire growth cone (Figure 4M). Thus, it appeared that the MTs were no longer being constrained to respect F-actin structures in a persistent manner. These observations, together with the increase in MT forward translocation rates, are consistent with more frequent transient decoupling of MTs from the F-actin network in the XMAP215 KD. We propose ‘transient decoupling’ rather than ‘permanent uncoupling’ because total disruption of F-actin leads to a MT exploration and translocation phenotype that is far more severe than the XMAP215 KD (not shown). Without the F-actin network, not only do MTs lose the ability to explore the entire growth cone space, resulting in much more MT track co-linearity than occurs in wild-type [38], but their plus-end velocities are also drastically reduced (not shown). Therefore, we suggest that XMAP215 functions to mediate the transient linkage regulation between MTs and F-actin, and so the knockdown biases MTs towards more frequent decoupling while still maintaining the ability to use F-actin as a guide.

The regulation of MT-F-actin interactions clearly plays a critical role within the growth cone, and there is a complicated interplay between these two cytoskeletal systems, which together, allows for directed axon outgrowth [1]. Yet, the mechanisms by which these interactions occur are only just beginning to emerge. It is notable that our CLASP screen, which identified XMAP215, also uncovered multiple F-actin interactors and MT-F-actin cross-linkers [6], and we previously observed that the fly orthologs of XMAP215 and CLASP genetically interact during axon guidance [6]. Discovering how XMAP215 is involved in mediating MT-F-actin coupling in both the axon to regulate retraction and the growth cone to control MT translocation, and whether this occurs through direct or indirect mechanisms such as by interacting with CLASP, will be a critical question for the future. Alternatively, it is conceivable that changes in MT dynamics may result from direct changes in MT-MT interactions in the XMAP215 KD, and this is another possible mechanism to examine in future studies.

## Conclusions

**Figure 5:**
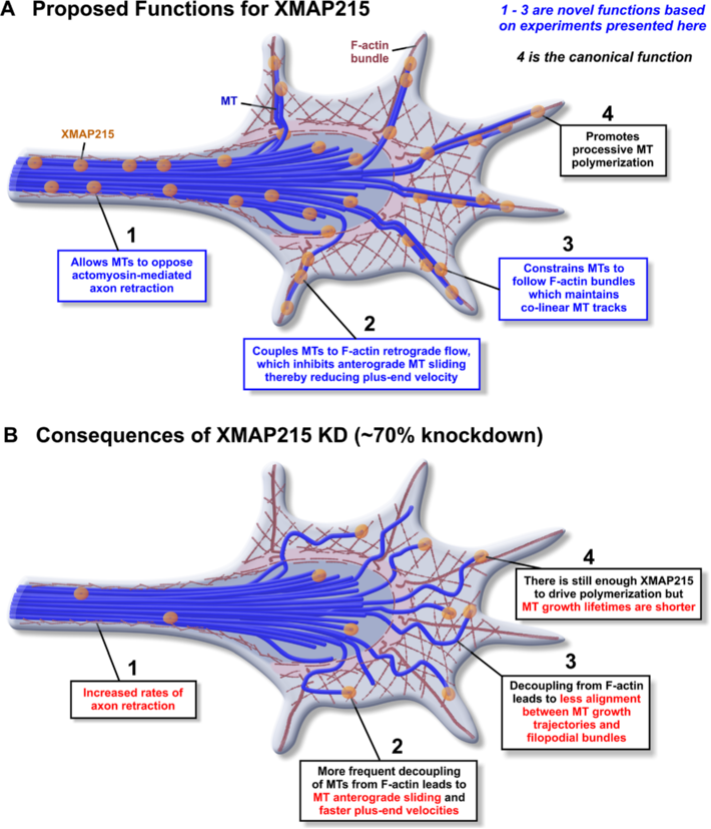
**Proposed model for XMAP215 function in neurons. (A)** Cartoon schematic of proposed functions of XMAP215 in wild-type conditions. Our data suggests that XMAP215 functions to mediate MT-F-actin coupling in both axons and growth cones. In the axon, as XMAP215 knockdown (KD) leads to a myosin II-dependent increase in axon retraction, this implicates XMAP215 as part of the machinery that stabilizes MT forces to oppose the actomyosin-mediated retraction (1). In the growth cone, XMAP215 KD leads to an increase in anterograde MT sliding (2) and a loss of colinearity of MT trajectories (3). As MT translocation rates and trajectory coherence within growth cones are strongly affected by coupling to F-actin retrograde flow, this data suggests that XMAP215 also contributes to the linkages between MTs and F-actin in the growth cone. These novel functions that we propose are in addition to the canonical function of XMAP215 - driving processive MT polymerization (4). **(B)** Consequences of XMAP215 KD. When XMAP215 is knocked down approximately 70%, this results in disruptions to all four functions, as described in the figure.

In this study, we identify new functions for the well-studied MT regulator XMAP215 in axon outgrowth and growth cone MT dynamics (summarized in Figure 5). While it was previously reported that axon outgrowth requires the mammalian ortholog, ch-TOG [12], here we provide unanticipated insights into XMAP215 function by using quantitative analysis of high-resolution time-lapse imaging of *Xenopus laevis* embryonic neurons. We demonstrate that reduced axon outgrowth after XMAP215 KD is due to an increase in spontaneous axon retractions and not an effect on axon outgrowth rates. As axon retraction is mediated by actomyosin contraction, this implicates XMAP215 as part of the machinery that stabilizes MT forces to oppose the actin-mediated retraction (Figure 5). Our most surprising discovery is that reducing XMAP215 functions leads to 20% faster MT plus-end velocities specifically within the growth cone. MT speckle microscopy data indicate that differences in overall MT translocation are a major contributor of this velocity change. As MT translocation rates within growth cones are strongly affected by coupling to F-actin retrograde flow, this data yet again suggests that XMAP215 contributes to the coupling between MTs and F-actin (Figure 5). Finally, our examination of MT tracks within growth cones after XMAP215 KD documents a striking change in MT behaviors, as MTs appear to no longer be constrained towards co-linearity. Thus, our analysis reveals that XMAP215 function and regulation may be significantly more complicated than previously appreciated. Rather than functioning solely as a MT polymerase, it is also involved in mediating MT-F-actin coupling in neurons, and our findings point to the importance of future investigations of XMAP215 function during MT and F-actin interactions.

## Methods

### Embryos

Eggs obtained from *Xenopus laevis* frogs (NASCO) were fertilized *in vitro*, dejellied, and cultured at 14 to 18°C in 0.1X Marc’s modified Ringer’s (MMR) using standard methods [39]. All experiments were approved by the Harvard Medical School Institutional Animal Care and Use Committee (IACUC) and performed according to national regulatory standards.

### Constructs and RNA

Capped mRNA was transcribed *in vitro* using SP6 or T7 mMessage mMachine Kit (Ambion, http://www.lifetechnologies.com/us/en/home/brands/ambion.html). RNA was purified (LiCl precipitation) and re-suspended in nuclease-free ddH20. Constructs: EB1-GFP pCS107 (gift from Danilchik lab), GFP-TOG (human, gift from Elisa Barbarese, University of Connecticut), XMAP215-GFP (gift from Hyman lab [10]), sub-cloned into pT7TS, mKate2-tubulin [40] and mKate2-EB1 in pCS2+. The dorsal blastomeres of embryos were injected four times fine at the two-to-four cell stage (in 0.1X MMR containing 5% Ficoll) with total mRNA amount per embryo: 100 to 250 pg EB1-GFP, 200 to 700 pg GFP-TOG, 700 pg XMAP215-GFP, 700 to 1,920 pg truncated XMAP215-GFP, 100 to 250 pg mKate2-EB1, 900 pg mKate2-tubulin for uniform labeling of MTs, or 360 pg mKate2-tubulin for speckle labeling.

### Morpholinos

Morpholino antisense oligonucleotides (MO) targeted to the translation start site of *Xenopus laevis* XMAP215 (5’-tcatccactcgctgtcatcccccat-3’, 5’-gcaatctgaggtcctgtttccgttc-3’) or standard control MO (5’-cctcttacctcagttacaatttata-3’) (purchased from Gene Tools, LLC, Philomath OR, USA) were injected into two-to-four cell stage embryos (4 to 11 ng/embryo). Knockdown was assessed by Western blot. Embryos at stage 35 to 36 were lysed in buffer (50 mM Tris pH 7.5, 5% glycerol, 0.2% IGEPAL, 1.5 mM MgCl_2_, 125 mM NaCl, 25 mM NaF, 1 mM Na_3_VO_4_, 1 mM DTT, supplemented with Complete Protease Inhibitor Cocktail w/EDTA (Roche, http://www.roche-applied-science.com/). For all growth cone experiments, individual embryos minus neural tubes were lysed and used for knockdown analysis. Blotting was carried out using rabbit polyclonal antibody to XMAP215 (gift of the Hyman lab, 1:2,500), with mouse anti-tubulin DM1alpha (Sigma, St. Louis MO, USA, 1:1,000) as a loading control. Detection was by chemiluminescence using Amersham ECL Western blot reagent (GE Healthcare Bio-Sciences, Pittsburgh PA, USA). The bands were quantified by densitometry using Photoshop (Adobe, San Jose CA, USA).

### Culture of Xenopus neural explants

Embryos were cultured in 0.1X MMR at 22°C to stage 22 to 24, and neural tubes were dissected as described [15,41]. Neural tube explants were plated in culture medium on laminin-coated (20 μg/mL) Mattek dishes, and axons and neural crest cells were imaged at room temperature 18 hours after plating, unless specified otherwise. For the blebbistatin experiments, 20 μM blebbistatin (with 0.02% DMSO) was added to the culture media immediately prior to time-lapse imaging.

### Immunocytochemistry

Whole-mount immunostaining was carried out using mouse anti-acetylated tubulin (Sigma, St. Louis MO, USA, T7451, 1:1,000), with goat anti-mouse Alexa Fluor 488 (Invitrogen, 1:1,000, http://www.lifetechnologies.com/us/en/home/brands/invitrogen.html) as a secondary antibody. Two-day-old embryos (incubated at 22°C) were fixed in Dent’s Fix for two hours at 22°C, rinsed in PBS, dehydrated in methanol and stored at −20°C. Embryos were processed for immunoreactivity by rehydrating and incubating in 1% triton-X 100, 1% DMSO in PBS for three hours, blocked (1% normal serum), then incubated in antibodies. Embryos were cleared in a 3:1 benzyl benzoate/benzyl alcohol solution and mounted on glass slides.

### Confocal microscopy

All live images were collected with a Yokogawa CSU-10 spinning disk confocal on a NikonTi-E inverted motorized microscope with a Nikon 60x Plan Apo 1.4 NA lens. Images were acquired with a Hamamatsu ORCA-AG cooled CCD camera controlled with MetaMorph (system purchased from RPI Co, Natick MA, USA and Micro Video Instruments Inc, Avon MA, USA). For timelapse, images were collected every two seconds for one to three minutes, using an exposure time of one second and 1x1 binning (except for GFP-TOG; mKate2-EB1, collected every five seconds for one minute, exposure time one second for 564 nm, two seconds for 488 nm).

For whole embryo immunostaining, images were collected with a Nikon A1R scan head on a Nikon Ti-E inverted microscope, using a 10x Plan Apo 0.45 NA lens (system purchased from RPI Co, Natick MA, USA and Micro Video Instruments Inc, Avon MA, USA). Images were acquired with photomultiplier tubes controlled with Nikon Elements. Z-series optical sections were collected with a step size of 5 μm, displayed as maximum z-projections. For all images, gamma, brightness, and contrast were adjusted on displayed images (identically for compared imaged sets) using ImageJ or Adobe Photoshop (Adobe, San Jose CA, USA).

### Phase contrast microscopy

Images were collected on a Nikon Ti inverted scope with a 20x Plan Apo 0.75 NA phase contrast objective lens, using a Hamamatsu ORCA-R2 cooled CCD camera controlled with MetaMorph (system purchased from RPI Co, Natick MA, USA). For timelapse, multi-point acquisition was used to capture multiple growth cones every five minutes for five hours. Images were compiled in ImageJ, and a Matlab script was written to manually record the x- and y- positions for each growth cone per timeframe, to measure axon outgrowth parameters.

### plusTipTracker software analysis

MT dynamics were analyzed from EB1-GFP movies using plusTipTracker [20]. The quality of the movies was assessed by examining comet detection and track linkage performance; movies were discarded if there were a large number of false negatives or false positives. The same parameters were used for all movies: maximum gap length, eight frames; minimum track length, three frames; search radius range, 5 to 12 pixels; maximum forward angle, 50°, maximum backward angle, 10°; maximum shrinkage factor, 0.8; fluctuation radius, 2.5 pixels.

### QFSM software analysis

Flow vectors were calculated using QFSM software [31]. To visualize F-actin flow, neuronal cultures were incubated in 3 nM kabarimide C conjugated to tetramethylrhodamine (TMR-KabC; gift from Tim Gomez) for three minutes, then washed with culture media, as done previously [42]. Masks of the growth cones were manually defined. For speckle detection, the confidence interval for statistical selection of speckles was 1e-06. For flow tracking, the following settings were used: integration window three frames, size range of the correlation templates 5 to 20 pixels, 20 pixels/frame maximum speed and 11 pixels minimum size. For speckle tracking, the search radius was ten pixels, and for flow analysis, the time window interpolation was three frames.

### Image analysis and statistics

Phenotypic quantification was typically performed blind of genotype and from multiple experiments to ensure reproducibility. Graphs were made in Microsoft Excel (Microsoft, Redmond WA, USA), and the Peltier Tech Box and Whisker Chart Utility for Excel was used to make box-plots. Unpaired two-tailed *t*-tests (GraphPad, La Jolla CA, USA) were used to determine statistical significance.

## Abbreviations

GFP: Green fluorescent protein; MO: Morpholino; MT: Microtubule; QFSM: Quantitative fluorescent speckle microscopy; +TIPs: Plus-end-tracking proteins; XMAP215 KD: XMAP215 knockdown.

## Competing interests

The authors declare that they have no competing interests.

## Authors’ contributions

LAL, GD and DVV designed the experiments. LAL, AS and AEF carried out the experiments and analyzed the data. LD wrote computer code for the QFSM rose-plot analysis. MAB and MWD provided unpublished reagents necessary for the study. LAL, GD and DVV wrote and edited the manuscript. All authors read and approved the final manuscript.

## Supplementary Material

Additional file 1 Time lapse montage demonstrates that XMAP215 KD leads to normal outgrowth velocities but increased rates of retraction (right panel), between 18 and 23 hours of culturing, compared to controls (left panel). Click here for file

Additional file 2 EB1-GFP comet velocity is increased in XMAP215 KD growth cones (right panel) compared to controls (left panel). Click here for file

Additional file 3 **A. Timelapse of *Xenopus *growth cone demonstrates XMAP215-GFP tracks MT plus-ends and also binds MT lattice within the growth cone and also in the axon shaft. B.** Timelapse of *Xenopus* growth cone demonstrates GFP-TOG localization to MT lattice within the growth cone and also in the axon shaft. **C.** Timelapse montage of a single MT, showing mKate2-EB1 (top panels) and GFP-TOG (bottom panels). This is an example where GFP-TOG does not track the plus-end and instead accumulates in relatively stable punctae along the MT lattice. Click here for file

Additional file 4 **A. Timelapse of mKate2-tubulin at low levels in control growth cone overlaid with flow vectors calculated by qFSM software. B.** Timelapse of mKate2-tubulin at low levels in XMAP215 KD growth cone overlaid with flow vectors calculated by qFSM software. **C.** Timelapse of F-actin speckles overlaid with flow vectors calculated by qFSM software in control growth cone. **D.** Timelapse of F-actin speckles overlaid with flow vectors calculated by qFSM software in XMAP215 KD growth cone. Click here for file
